# Total Selenium Level and Its Distribution between Organs in Beef Cattle in Different Selenium Status

**DOI:** 10.3390/ani13243885

**Published:** 2023-12-18

**Authors:** Marta Juszczak-Czasnojć, Agnieszka Tomza-Marciniak, Bogumiła Pilarczyk, Dariusz Gączarzewicz

**Affiliations:** Department of Animal Reproduction Biotechnology and Environmental Hygiene, Faculty of Biotechnology and Animal Husbandry, West Pomeranian University of Technology in Szczecin, Klemensa Janickiego 29, 71-270 Szczecin, Poland; agnieszka.tomza-marciniak@zut.edu.pl (A.T.-M.);

**Keywords:** beef, cattle, distribution, selenium, tissues

## Abstract

**Simple Summary:**

Selenium is a trace element needed for proper growth and development in both humans and animals. The aim of this study was to determine the concentration of total selenium in the main tissues and to evaluate the differences in tissue distribution in beef cattle with different selenium status. It was found that despite receiving supplementation, 44.44% of the examined cattle were deficient in selenium. The highest percentage of deficient animals was recorded among the heifers (62.5%), and the lowest in bulls (30.43%). Differences in Se tissue distribution were observed between Se-deficient animals and those with normal Se status. The organs most susceptible to Se deficiency are the semitendinosus muscle, lungs, heart and liver. Our findings confirm that Se deficiencies can be found among supplemented animals. Selenium deficiency in beef cattle may be related to the uneven uptake of the element, but also to the individual predispositions of animals. It is recommended to monitor Se levels to detect deficiencies and, if necessary, to respond by increasing selenium supply.

**Abstract:**

The aim of this study was to determine the Se concentration in the main tissues of beef cattle and to evaluate the differences in tissue distribution between animals with different selenium status. Selenium concentration was determined in the serum, longissimus dorsi muscle, semitendinosus muscle, kidney, heart, liver, spleen and lungs of cows, heifers and beef bulls, using spectrofluorimetric method. Despite receiving supplementation, 55.6% animals demonstrated an optimal Se level, while 44.4% were deficient. The mean serum Se concentration was significantly higher (*p* < 0.05) in animals with a normal Se status than in Se-deficient animals. Differences in Se tissue distribution were observed between Se-deficient animals and those with normal Se status. The organs most susceptible to Se deficiency are the semitendinosus muscle, lungs, heart and liver. In both normal and Se-deficient animals, significantly higher Se concentrations were observed in the kidney than other organs (*p* < 0.05), and the lowest in the muscles. As Se deficiencies can be found among supplemented animals, the level of Se should be monitored in beef cattle in order to detect possible Se deficiencies, which may have negative health effects for animals and reduce the value of animal products as a source of Se in the human diet.

## 1. Introduction

Selenium plays a number of enzymatic and structural roles in the body through its incorporation into selenoproteins [1].

The tissue distribution of selenium in the mammalian body depends primarily on its chemical form and the total dietary intake. Selenium is best absorbed in its organic form, i.e., selenomethionine (SeMet), this being a better source of Se than inorganic compounds, such as sodium selenite or selenate. In ruminants, around 60% of SeMet is incorporated into the protein of rumen microorganisms, with an intestinal digestibility of about 80% [2,3,4]. Dietary Se is absorbed in the intestine and then transported in the blood from the intestine to the liver, where it is reduced to selenide and delivered to the tissues and target organs via blood. It is then incorporated into specific selenoproteins. The largest amounts of Se are found in blood cells, liver, spleen and heart tissue. In addition, Se deficiency is first noted in the heart, skeletal muscles and liver [5].

The key storage organ for Se, and the best indicator of its deficiency, is the liver. Where dietary intake is adequate, approximately 30% of the total Se contained in tissues is found in the liver, followed by 30% in the muscles, 15% in the kidneys and 10% in plasma [6,7,8]. Selenium is mainly excreted in the urine (50–70%), and to a lesser extent, in the faeces and exhaled air; however, respiratory Se excretion only occurs when Se intake is high [9]. The kidneys play a significant role in regulating the amount of Se in the body and are of particular importance in the case of Se deficiency: when the supply of Se is insufficient, Se excretion in the urine is limited [9]. Higher concentrations are also noted in the kidneys than in the liver in cases of Se deficiency; this has been attributed to the fact that the kidneys have a minimal demand for Se when the supply is low [7].

The highest retention rate in Se-deficient animals is found in the thyroid gland and in the reproductive organs, i.e., in the testes and ovaries, where Se has been found to accumulate during periods of low supply; these organs have priority in selenium uptake over the liver, heart, skeletal muscles and erythrocytes. Hence, the highest selenium levels are present in inedible tissues, which is unfavourable from the point of view of the consumer [10,11]. Akahoshi et al. [12] report a gradual decrease in Se content in the heart, liver, lungs, kidneys, pancreas and spleen in rats fed low-Se feed, while the Se concentration in the testes remained at the same level. However, in mice with an excess of SeMet, a rapid increase in Se content was observed in all organs [12].

Particularly low concentrations of selenium are found in ruminants [13,14]. This is partly due to the fact that ruminants consume plants, which accumulate varying levels of Se [15], and animal premixes contain inorganic Se, which is only 13% absorbed [16]. Furthermore, a large proportion of dietary Se is reduced to insoluble forms by rumen microbes [17,18]; this is especially true of inorganic selenium. In addition, ruminants demonstrate much lower absorption of inorganic Se than monogastric animals, probably due to the action of ruminal microorganisms [19], which convert sodium selenite to insoluble forms; however, these microbes demonstrate five-times greater uptake and absorption of SeMet [20,21]. This has been attributed to the fact that organic selenium undergoes fewer changes in the rumen, making it more bioavailable [3,20,22].

According to Juniper et al. [22], both the source of Se and the concentration of Se in the diet affect the total concentration of Se and its distribution in animal tissues. In beef cattle supplemented with selenium yeast, significant increases in Se concentration were observed in various tissues and organs, such as skeletal muscles (longissimus dorsi and musculus psoas major), liver, heart and kidney, compared to non-supplemented animals; these values were 26, 16, 8 and 3 times higher, respectively, compared to the non-supplemented group. The authors also observed differences in the tissue distribution of Se between groups of animals: non-supplemented controls, i.e., with a low Se status (0.225 µg Se/mL in blood), demonstrated the highest Se level in the kidneys, while those receiving supplementation mostly accumulated Se in the liver [22]. This is in line with research by Cristaldi et al. [18], who observed higher concentrations in the liver than the kidneys at higher Se intakes and that Se concentration in the organs increased with dietary concentration.

Numerous studies on ruminants confirm that maintaining the appropriate level of Se improves the utilization of nutrients from feed, as manifested by increased daily weight gain in bulls and milk production in dairy cows. This is due to the increased microbial population and enzyme activity in the rumen [23,24,25].

Excess selenium in cattle causes a disease called selenosis. It manifests itself by decreased appetite, apathy, hair loss, lameness and deformation of the stratum corneum of the hoof. It may lead to damage to the joints of the limbs, degeneration of the heart muscle and liver cirrhosis [26].

In cattle, Se deficiency is not only a problem associated with increased animal morbidity and weaker growth, but also with a reduced ability to meet consumer demand for the element. The main source of Se in the human diet is products of animal origin, and hence Se deficiency in the tissues and organs of farm animals, including cattle, is associated with reduced Se supply in humans [27].

Recent years have seen an increase in food awareness among consumers and, greater expectations regarding the food sector. Consumers are increasingly willing to choose functional foods that have a positive effect on the body. Of all meats, beef offers the greatest nutritional value, and the presence of suitable amounts of microelements, including Se, in raw and processed food ensures their optimal intake [28]. The daily requirement for Se for human is 10–15 μg in the first year of life and increases to 45–70 μg after the age of 18, depending on the physiological state [29].

Although many studies have been conducted on the tissue distribution of Se in various animal species [30,31], there is little data on how it is affected by dietary Se status in beef cattle [8,22]. The present study is the first to be carried out in Poland, an area known to suffer from Se deficiency.

The aim of the study was to determine the concentration of total selenium in the main tissues and to evaluate the differences in tissue distribution in beef cattle with different selenium status.

## 2. Materials and Methods

### 2.1. Animals

The research material consisted of tissues and organs of beef cattle kept in a conventional semi-intensive system in the Zachodniopomorskie Voivodeship. The animals were owned by an agricultural company whose activity covers the entire production cycle: from the production of fodder and animal husbandry to slaughter and production of food products of animal origin.

The animals were kept in accordance with the European Convention for the Protection of Vertebrate Animals [32], and met the conditions of the Act of 29 June 2007, in force in Poland [33], and the Minister of Agriculture and Rural Development Regulation of 10 September 2015, on the minimum conditions for keeping farm animal species, valid since 1 January 2018 [34].

The study included material from 63 animals: 24 cows (mean age 83, range 50–146 months), 16 heifers (mean age 22, range 19–24 months) and 23 bulls (mean age 23, range 21–25 months). The group included the following breeds: Simmentaler, Salers, Charolais, Hereford, Limousine and Red Angus, as well as crossbreeds of meat breeds (others). The average live weight of the animals was for a cow 645 kg, bulls 758 kg and heifers 452 kg. The cows were dry.

The cattle remained on pasture from spring (March) to late autumn (October/November). During the winter, they received haylage and hay harvested in the summer, plus pomace and corn. The material for the study was collected in the winter (February). The daily nutritional ration for each individual was as follows:

(a) Cows: 25 kg of haylage, 6–8 kg of pomace, 0.1 kg of mineral feed containing 12 mg/kg of inorganic Se and 8 mg/kg of organic Se, bulk straw and mineral lick containing 10 mg of sodium selenium/kg.
(b) Heifers: 14–16 kg of haylage, 2 kg of concentrated feed, 6–8 kg of bagasse, as much straw as needed and a mineral lick containing 10 mg of sodium selenium/kg.
(c) Bulls: 8 kg of maize, 6 kg of haylage, 6–8 kg of wet feed based on stillage (20% protein, 7.5 MJ), 4–6 kg of beet pulp, straw as desired and a mineral lick containing 10 mg of sodium selenium/kg.

Total nutritional compositions of the rations are presented Table 1.

### 2.2. Sample Collection

The research material was collected during slaughter, with the consent of the District Veterinary Officer (no. PIW.HP.9260/Uppz/Bad/1/2019) and under the supervision of the veterinarian present at the time of slaughter. The slaughter was planned by the farm and no animal was slaughtered specifically for these tests.

Liver, kidney (cortical and medullary layers), lung, spleen, heart, semitendinosus muscle, longissimus dorsi muscle and blood were collected. Tissues and organs were collected in plastic bags, and blood was collected in a dry tube without anticoagulant. Each sample was labelled, chilled immediately and transported to the laboratory. In the laboratory, tissues and organs were cleaned with saline and homogenized using a laboratory tissue homogenizer. The blood was centrifuged to separate the serum. Samples prepared in this way were stored at −20 °C for analysis. Selenium status was established based on serum Se concentration of the animals, according to the criteria proposed by Puls [35]. For comparative analysis, a division into two groups was used, i.e., normal Se concentration: 0.08–0.3 µg/mL and deficient concentration: below 0.08 µg/mL.

### 2.3. Ethical Consent

According to Polish Journal of Laws [36] 2015, item 266, Act on the protection of animals used for scientific or educational purposes, killing animals for the purpose of using their organs or tissues is not regarded as a procedure and hence does not require ethics committee approval (Article 1 point 2), especially since the killing of animals resulted from the typical agricultural activity carried out by the enterprise related to animal production (Article 2 point 6, article 3).

The animals were delivered to the slaughterhouse and slaughtered in accordance with the applicable regulation: Council Regulation (EC) No. 1/2005 [37] and Council Regulation (EC) No. 1099/2009 [38].

### 2.4. Chemical Analyses

#### 2.4.1. Reagents

Most chemicals were obtained from Chempur^®^ (Piekary Śląskie, Poland), except 2,3-diaminonaphtalene (DAN), which was obtained from Sigma Aldrich (St. Louis, MO, USA). All chemicals used were of analytical reagent grade. The certified reference materials were Seronorm (Trace Elements Serum L-1) (Sero AS, Billingstad, Norway), obtained from Sero (Billingstad, Norway), and BCR 185R (bovine liver). Mean Se concentration obtained 92% of the reference values.

#### 2.4.2. Se Determination

Se concentrations in serum, liver, kidney, lung, spleen and the heart, as well as the semitendinosus and longissimus dorsi muscles, were determined using spectrofluorometric methods. The samples were digested in HNO_3_ at 230 °C for 180 min and in HClO_4_ at 310 °C for 20 min. Then, selenate (Se^6+^) was reduced to selenite (Se^4+^) using 9% HCl. Selenium concentration was determined with 2,3–DAN under conditions of controlled pH (pH 1–2) via the formation of selenodiazole complex. This complex was extracted into cyclohexane. EDTA and hydroxylamine hydrochlorine were used as masking agents. Se concentration was determined fluorometrically using a Shimadzu RF–5001 PC spectrofluorophotometer (Shimadzu, Duisburg, Germany). The excitation wavelength was 376 nm, and the fluorescence emission wavelength was 518 nm.

### 2.5. Statistical Analysis

Statistical analysis was performed using Statistica software (StatSoft Inc., ver. 13.3 StatSoft, Tulsa, OK, USA). The normality of variable distribution was tested with the Shapiro–Wilk test. Variables with a non-normal distribution were then adjusted to a normal distribution via logarithmic transformation and then subjected to one-way analysis of variance. Correlation between analysed tissues was assessed using Pearson R Correlation (Supplementary Tables S1–S8). Significant differences were determined using Duncan’s test. Differences were considered statistically significant at a *p*-value of <0.05.

## 3. Results

In all animals, the serum Se content fell within the range of 0.019 to 0.167 μg/mL. A total of 35 out of 63 (55.56%) animals demonstrated an optimal Se level (0.08–0.3 μg/mL), while 28 out of 63 (44.44%) were deficient (Table 2).

In both Se-deficient and normal animals, significantly higher Se concentrations were observed in the kidney (*p* < 0.05), where the mean concentration was 1.526 µg/g in animals with normal Se levels and 1.313 µg/g in animals with Se deficiency. In all animals, regardless of Se status, the lowest Se concentration was found in the muscles: 0.114 µg/g in the longissimus dorsi muscle and 0.111 µg/g in the semitendinosus muscle in animals with normal Se levels, and 0.09 and 0.069 µg/g, respectively, in animals diagnosed with Se deficiency.

As the liver is a storage organ for Se, and hence receives a considerable amount of Se for accumulation during absorption, changes in dietary Se intake are quickly reflected in liver Se concentration. However, more surprising results were obtained for the tested muscles. Our research shows that the semitendinosus muscle is more exposed to Se deficiency than the longissimus dorsi muscle. Animals with Se deficiency demonstrated significantly lower Se content in the semitendinosus muscle (*p* < 0.05) than those with normal Se levels (Table 2). The longissimus dorsi muscle seems to be more resistant to changes in Se concentration than the semitendinosus muscle. Examining all animals together (Table 2), it was noted that the Se concentration in the longissimus dorsi muscle did not differ significantly (*p* = 0.05) between those with optimal and deficient Se levels.

Between the two groups of animals, the only organs demonstrating comparable Se concentrations were the spleen and kidney; these differences were not statistically significant (*p* > 0.05) (Table 2).

In general, the tissue distribution of Se in animals in both groups was as follows: kidney > liver > spleen > heart > lungs > longissimus dorsi muscle > semitendinosus muscle.

The *p* value given in the table refers to differences in the same tissues between two analysed group of animals.

Cows.

A total of 13 out of 24 (54.17%) animals had an optimal Se level, while 11 out of 24 (45.83%) were deficient (Table 3).

In both groups of animals, the highest Se levels were observed in the kidney (*p* < 0.05), where the mean concentration was 1.375 µg/g in animals with normal Se levels and 1.052 µg/g in deficient animals. The lowest mean Se concentrations were in the muscles: 0.113 µg/g in the longissimus dorsi muscle and 0.119 µg/g in the semitendinosus muscle in the normal Se group, and 0.063 and 0.055 µg/g, respectively, in the Se-deficient animals.

Only the spleen and kidney demonstrated similar Se concentrations between the normal and Se-deficient groups (*p* > 0.05) (Table 3).

The tissue distribution of Se differed slightly between normal and Se-deficient animals. In normal animals, the Se concentration followed the sequence kidney > liver > spleen > heart > lungs > semitendinosus > longissimus dorsi muscle; in Se-deficient animals, the sequence was kidney > liver > spleen > heart > lungs > longissimus dorsi muscle > semitendinosus muscle (Table 3).

The *p* value given in the table refers to differences in the same tissues between two analysed group of animals.

In the case of cows with optimal Se status, no significant differences in Se concentration were observed between the longissimus dorsi muscle and the semitendinosus muscle. In the case of Se-deficient cows, the Se concentration was significantly higher in the longissimus dorsi muscle than in the semitendinosus muscle. It was shown that the age of cows and the number of parities had no effect on the Se content in the cows (r = 0.092, *p* = 0.676 and r = 0.270, *p* = 0.224).

Bulls.

In bulls, serum Se concentration ranged from 0.019–0.166 μg/mL. A total of 16 out of 23 (69.57%) animals had an optimal level while 7 out of 23 (30.43%) were deficient (Table 4).

Comparing the normal and Se-deficient animals, significant differences in Se level (*p* < 0.05) were only observed in the heart. The lowest Se concentration was found in the muscles. The mean Se concentrations in the longissimus dorsi muscles and semitendinosus muscles were 0.121 and 0.108 µg/g, respectively, in animals with normal Se levels, and 0.118 and 0.085 µg/g, respectively, in animals diagnosed with Se deficiency (Table 4).

Differences were observed in the overall tissue distribution of Se with regard to Se status. These differences affected the heart, lungs and spleen. In animals with a normal Se status, the Se concentration was arranged in the following order: kidney > liver > spleen > heart > lungs > longissimus dorsi muscle > semitendinosus muscle. For Se-deficient animals, the order was kidney > liver > lungs > spleen > heart > longissimus dorsi muscle > semitendinosus muscle.

The *p* value given in the table refers to differences in the same tissues between two analysed group of animals.

Heifers.

In heifers, the serum Se concentration ranged from 0.033–0.127 μg/mL. A total of 6 out of 16 (37.5%) animals were found to have normal Se levels, while 10 out of 16 (62.5%) were deficient.

The lowest Se concentration was found in the muscles. The mean Se concentrations in the longissimus dorsi muscles and semitendinosus muscles were 0.106 and 0.1 µg/g, respectively, in animals with normal Se levels, and 0.101 and 0.076 µg/g, respectively, in animals diagnosed with Se deficiency. In both groups of animals, the highest Se levels were observed in the kidney (*p* < 0.05). Of the tested organs, only the liver demonstrated significant differences in Se content between the two groups of animals (*p* < 0.05) (Table 5).

The tissue distribution of Se differed between heifers with optimal and deficient selenium status, with differences observed in the liver, spleen, heart and lungs. In animals with a normal Se status, the Se concentration was arranged in the following order: kidney > liver > spleen > lungs > heart > semitendinosus > longissimus dorsi muscle. In contrast, in Se-deficient animals, it was arranged as kidney > spleen > liver > heart > lungs > longissimus dorsi muscle > semitendinosus muscle.

The *p* value given in the table refers to differences in the same tissues between two analysed group of animals.

The bulls demonstrated the highest Se levels in all tested tissues, except the spleen. All statistically significant differences between animal organs within each group are presented in Table 6. The correlation between Se concentrations in serum and individual organs within a group with normal and deficient Se status was determined using Pearson’s correlation coefficient. A significant correlation was found between the Se content of the serum, liver and heart of bulls with deficient Se status. The correlation coefficients between serum and individual organs within a group are presented in Table 7.

**Figure 1:**
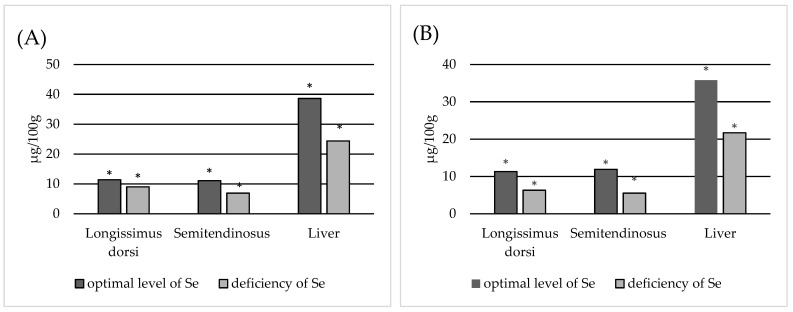

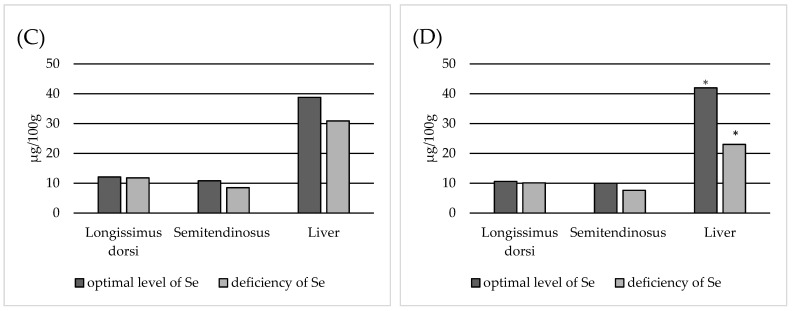
Estimation of Se intake from liver and muscles (100 g). (**A**) obtained Se; (**B**) Se obtained from cows; (**C**) Se obtained from bulls; (**D**) Se obtained from heifers; *—statistically differences (*p* < 0.05) in Se concentration in tissue between animals with normal and Se-deficient status.

Among the edible animal tissues, the most important are the muscles and liver. Compared to the kidney, which contains the highest levels of Se, the liver contains around four times less Se, and the muscles about 13 times less. The estimated Se uptake from these tissues per 100 g of product is shown in Figure 1. Data analysis shows that in addition to being susceptible to decreases in dietary Se intake, the liver appears to contain approximately 1.5 times more Se in animals with a normal selenium status than in Se-deficient animals. A similar situation is noted in the case of the semitendinosus muscle, especially in relation to cows, and animals in general. Considering that the concentration of Se in muscles is generally low and can be significantly reduced in the semitendinosus muscle in Se-deficient animals, this is not beneficial for the consumer.

## 4. Discussion

The European diet has long been known to be deficient in selenium [29] due to the presence of low Se concentrations in the soil in the region. Experiments conducted in Finland, New Zealand and Great Britain have shown that the use of mineral fertilizers enriched with selenium salts increases the content of Se in crops and thus in the diet of animals and humans [39]. However, despite the great importance of Se in ensuring the proper development of human and animal organisms, no attempts have been made to address this problem on the national level, e.g., by legislating to increase the content of this element in fertilizers. This is largely due to the risk of soil and water contamination, as was the case in Finland [39].

Although pork and poultry are more popular than beef in Europe, recent years have seen an increase in the popularity of beef products, and there is a widely held belief that beef products are healthier and have better nutritional properties than other types of meat. According to the Polish Central Statistical Office, the consumption of beef in Poland rose from 1.2 kg per capita per year in 2015 to 4.1 kg in 2019 [40,41]. It is predicted that beef consumption will rise to 14 kg per capita in the years 2024–2027, accounting for 17.5% of total meat consumption [42].

Due to its basic chemical composition and exogenous content, beef is a valuable source of protein with high biological value and a ready source of B vitamins (B2, B3, B5 and B6) and minerals (e.g., iron, phosphorus, zinc or selenium) [43].

Unfortunately, due to the nutritional status of pasture in Europe and the poor assimilation of selenium from plants by ruminants [44,45], beef cattle are often deficient in Se. Indeed, Se deficiency was noted in 44.4% of the animals tested in the present study, despite all being fed in the same way while also receiving Se supplementation. This could be due to uneven feed intake by individual animals, inaccurate mixing, or the individual characteristics of animals, all of which can affect the course of Se metabolism.

Pereira et al. [46] found the mean Se content in the serum of Galician Blonde bulls (GB) to be 0.268 μg/mL (range 0.181–0.408). This value exceeds our present values for all groups, regardless of breed. In the present study, the highest serum concentration of Se was found in cows with a normal Se status (0.126 µg/mL), this value being half that identified by Pereira et al. [46], who also report that the tissue distribution of Se in animals was brain > kidney > spleen > liver > semitendinosus muscle, which was similar to that of the Se-deficient heifers in the present study (kidney > spleen > liver > heart > lungs > longissimus dorsi muscle > semitendinosus muscle). However, a different distribution was noted in the remaining groups (Table 3 and Table 4), i.e., bulls with a normal Se status and cows were characterised by a distribution of kidney > liver > spleen > heart > lungs > semitendinosus muscle > longissimus dorsi muscle.

In a study of Limousine beef cattle from an organic farm, Biel et al. [47] found the highest Se content to be in the kidneys and liver (1.39 and 0.39 µg/g DM) and the lowest in the heart and musculus semitendinosus (0.21 and 0.24 µg/g DM). In the present study, the highest Se value in the studied Limousine cattle was also recorded in the kidneys and liver (1.376 and 0.343 µg/g), and the lowest in the muscles. Moreover, in calves, Biel et al. [47] report the highest content to be in the kidneys and heart (0.80 and 0.32 µg/g DM), and the lowest in the liver and musculus semitendinosus (0.226 and 0.065 µg/g DM), with the following tissue distributions: kidney > liver > semitendinosus > heart > tongue > brain for cows and kidney > heart > liver > brain > musculus semitendinosus > tongue for calves. However, our present findings indicate that cattle with normal and Se-deficient statuses had the same Se distribution: kidney > liver > spleen > heart > lungs > longissimus dorsi muscle > semitendinosus muscle. These similarities between our present findings and those of Biel et al. [47] may be due to the fact that the studies included the same breed of cattle and that the animals were kept under similar conditions and in the same geographical area.

Ullah et al. [48] noted that liver, heart and skeletal muscles are most sensitive to Se deficiency; however, these were found to be the liver, heart, longissimus dorsi and semitendinosus muscles in the present study. Also, in our study, significant differences were found between animals with normal Se status and Se deficiency (Table 2, Figure 1).

Protein profiling studies by Clerens et al. [49] indicated that while the overall protein profiles were similar for four muscle types, significant differences in intensity (*p* < 0.05) were observed between 24 protein spots taken from the muscles. Recently, special attention has been paid to the presence of selenoprotein N in muscles and its role in the pathogenesis of some muscle diseases. Selenoprotein deficiency or malfunction is associated with the occurrence of weakness of the torso and neck muscles, or rigid spine muscular dystrophy [50].

Other organs with high Se content are the spleen, heart, testes and lungs [48]. Our present findings confirm the presence of significant differences in Se content between normal and Se-deficient animals, with optimal Se concentrations being observed in the heart and lungs (Table 2), which are susceptible to Se deficiency. Ensuring that their Se level remains optimum is a priority due to their essential functions [51,52].

Lawler et al. [53] report that both the dose of dietary Se and its form may influence its tissue distribution in cattle. The authors note that after administration of wheat enriched with organic Se, Se concentration increased considerably in the kidneys, spleen, semitendinosus muscle and the hair, but remained constant in the liver. However, after administration of sodium selenite, the greatest increase in concentration was observed in the kidneys and hair, and a significant increase was noted in the semitendinosus muscle. In addition, cattle fed sodium selenate had higher liver Se concentrations than those fed grass high in Se. While the animals in the present study also received selenium supplements, this was provided mainly as inorganic Se, which is assimilated to a lesser extent; as a result, some Se deficiencies were noted.

Numerous studies show that selenium deficiency is quite common in cattle. Salwa et al. [54] found that cows fed feed without additional Se were characterized by low Se levels, i.e., below 0.026 µg/mL serum. In the present study, the Se concentration in cows with Se deficiency was 0.038 µg/mL. Balicka-Ramisz et al. [55] report that 23.6% of dairy cows in the northern part of Poland are characterized by marginal Se concentrations (mean: 0.041 µg/mL). In turn, 39.3% showed a threshold value, ranging from 0.041 to 0.079 µg/mL. Only 37.1% demonstrated normal Se concentrations. In addition, Andrzejewski [56] found Simmental cows not supplemented with selenium to have low mean serum Se concentrations (0.037 µg/mL). Similar values were also observed in animals that received Se preparations in the present study.

Low Se concentrations are also observed in both Europe and the USA. For example, Pavlata et al. [57] report that about 42% of cows, 80% of calves, 100% of heifers and 90% of fatteners in the Czech Republic show a marginal level of Se in the body. In the present study, 62.5% of heifers were found to be deficient in Se. Davy et al. [58] report that selenium deficiency is common in cattle in California, with a mean selenium level of 0.18 mg/mL. In addition, lower levels of Se are observed in animals in Europe, which is likely due to the lower level of selenium in the soil in Europe compared to the USA [6,41,59,60].

## 5. Conclusions

It was found that despite receiving supplementation, 44.44% of the examined cattle were deficient in selenium. The highest percentage of deficient animals was recorded among the heifers (62.5%), and the lowest in bulls (30.43%). Differences in Se tissue distribution were observed between Se-deficient animals and those with normal Se status. The organs most susceptible to Se deficiency are the semitendinosus muscle, lungs, heart and liver; in these organs, a significantly lower (*p* < 0.05) concentration of Se was observed in the deficient animals. This entails a number of adverse effects for the consumer. It has been shown that in animals with a normal selenium status, the liver provides 1.5 times more Se than the liver of Se-deficient animals. A similar situation is noted in the case of the semitendinosus muscle, especially from cows, but also animals in general. Therefore, there is a need for further measures aimed at increasing the Se content in animal tissues intended for consumption. As Se deficiencies still can be found among supplemented animals, it is recommended that the level of Se should be monitored in beef cattle in order to detect possible Se deficiencies, and to take prompt action to prevent the negative health effects associated with Se deficiencies in animal products. For cows that have received mineral feed, it is recommended to use Se in organic form instead inorganic Se. In the feeding of bulls and heifers, the feed ration should be recomposed, with Se as an ingredient.

## Figures and Tables

**Table 1:** The total chemical composition of the feedstuffs (mean ± standard deviation).

Chemical Composition	Cows	Heifers	Bulls
Dry matter (g)	10,200 ± 158.1	8370 ± 103.4	11,900 ± 115.8
Crude protein (g)	802.1 ± 68.7	761.3 ± 54.5	1331.2 ± 98.3
Raw fibre (g)	1628.2 ± 111.2	1524.7 ± 95.8	2387.2 ± 167.7
Metabolic energy (MJ)	93.28 ± 14.6	79.24 ± 7.4	115.74 ± 19.2
Raw ash (%)	6.34 ± 0.35	5.95 ± 0.51	8.29 ±0.07
Selenium (mg)	3.06 ± 0.02	3.08 ± 0.05	2.52 ± 0.1
Selenium (mg/kg)	0.09 ± 0.03	0.12 ± 0.06	0.01 ± 0.01

**Table 2:** Se concentration in individual tissues of animals with normal and deficient Se status.

Tissue		Se Concentration (µg/g w.w.)		Anova *p* Value	Normal Range [33]
		Optimal Se Level	Deficient Se Status		
Serum μg/mL	Mean	0.121 ^abcde^	0.053 ^abcde^		
	Sd	0.024	0.020	<0.01	0.08–0.3
	Range	0.081–0.167	0.019–0.079		
Longissimus dorsi muscle	Mean	0.114 ^fghij^	0.09 ^fgh^		0.07–0.15
	Sd	0.049	0.044	0.05	(muscle)
	Range	0.028–0.232	0.027–0.182		
Semitendinosus muscle	Mean	0.111 ^klmno^	0.069 ^ijkl^		0.07–0.15
	Sd	0.031	0.036	<0.01	(muscle)
	Range	0.042–0.171	0.021–0.158		
Kidney	Mean	1.526 ^afkprst^	1.313 ^afimnop^		
	Sd	0.463	0.413	0.10	1–1.5
	Range	0.862–2.47	0.452–1.966		
Lungs	Mean	0.215 ^bglpu^	0.165 ^bjm^		
	Sd	0.090	0.072	0.01	
	Range	0.118–504	0.045–0.273		
Heart	Mean	0.223 ^chmrw^	0.158 ^cn^		
	Sd	0.061	0.076	<0.01	
	Range	0.107–0.337	0.039–0.293		
Spleen	Mean	0.223 ^dinsz^	0.217 ^dgko^		
	Sd	0.110	0.104	0.19	
	Range	0.100–0.462	0.037–0.435		
Liver	Mean	0.386 ^ejotuwz^	0.244 ^ehlp^		
	Sd	0.136	0.141	<0.01	0.25–0.5
	Range	0.127–0.713	0.063–0.590		

w.w.—wet weight. lowercase letters—the same lowercase letters indicate statistically significant differences at *p* < 0.05 between animal tissues within a one group of animals: normal and deficiency selenium level.

**Table 3:** Se concentration in individual tissues in cows.

Tissue		Se Concentration (µg/g w.w.) in Cows		Anova *p* Value
		Optimal Se Level	Deficient Se Status	
Serum μg/mL	Mean	0.126 ^ab^	0.038 ^bc^	
	Sd	0.027	0.014	<0.01
	Range	0.081–0.167	0.023–0.075	
	25–95%CI	0.128–0.142	0.039–0.047	
Longissimus dorsi muscle	Mean	0.113 ^cd^	0.063 ^def^	
	Sd	0.048	0.036	0.01
	Range	0.043–0.232	0.027–0.157	
	25–95%CI	0.118–0.142	0.066–0.087	
Semitendinosus muscle	Mean	0.119 ^ef^	0.055 ^ghi^	
	Sd	0.039	0.030	<0.01
	Range	0.042–0.171	0.021–0.117	
	25–95%CI	0.123–0.143	0.058–0.075	
Kidney	Mean	1.375 ^aceghij^	1.052 ^adgjklm^	
	Sd	0.435	0.319	0.07
	Range	0.908–2.397	0.452–1.414	
	25–95%CI	1.418–1.667	1.085–1.280	
Lungs	Mean	0.184 ^gk^	0.122 ^j^	
	Sd	0.061	0.067	0.03
	Range	0.119–0.285	0.045–0.258	
	25–95%CI	0.190–0.221	0.129–0.167	
Heart	Mean	0.214 ^hl^	0.137 ^k^	
	Sd	0.067	0.086	0.03
	Range	0.128–0.337	0.039–0.291	
	25–95%CI	0.220–0.257	0.145–0.195	
Spleen	Mean	0.255 ^i^	0.199 ^behl^	
	Sd	0.120	0.081	0.22
	Range	0.1–0.416	0.037–0.353	
	25–95%CI	0.267–0.336	0.207–0.254	
Liver	Mean	0.358 ^bdfjkl^	0.217 ^cfim^	
	Sd	0.160	0.153	0.04
	Range	0.127–0.713	0.063–0.575	
	25–95%CI	0.372–0.454	0.232–0.319	

w.w.—wet weight. lowercase letters—the same lowercase letters indicate statistically significant differences at *p* < 0.05 between animal tissues within a one group of animals: normal and deficiency selenium level.

**Table 4:** Se concentration in individual tissues in bulls.

Tissue		Se Concentration (µg/g w.w.) in Bulls		Anova *p* Value
		Optimal Se Level	Deficient Se Status	
Serum μg/mL	Mean	0.123 ^ab^	0.066 ^ab^	
	Sd	0.022	0.022	<0.01
	Range	0.092–0.166	0.019–0.079	
	25–95%CI	0.125–0.134	0.069–0.087	
Longissimus dorsi muscle	Mean	0.121 ^cd^	0.118 ^cd^	
	Sd	0.044	0.032	0.86
	Range	0.058–0.208	0.073–0.158	
	25–95%CI	0.125–0.144	0.122–0.147	
Semitendinosus muscle	Mean	0.108 ^ef^	0.085 ^ef^	
	Sd	0.026	0.032	0.07
	Range	0.079–0.166	0.034–0.13	
	25–95%CI	0.110–0.122	0.089–0.114	
Kidney	Mean	1.557 ^aceghij^	1.564 ^aceghij^	
	Sd	0.445	0.393	0.97
	Range	0.862–2.407	0.096–1.966	
	25–95%CI	1.594–1.79	1.614–1.927	
Lungs	Mean	0.233 ^gk^	0.204 ^g^	
	Sd	0.099	0.075	0.48
	Range	0.133–0.504	0.078–0.273	
	25–95%CI	0.241–0.286	0.213–0.273	
Heart	Mean	0.234 ^hl^	0.183 ^h^	
	Sd	0.044	0.070	0.05
	Range	0.163–0.316	0.042–0.256	
	25–95%CI	0.237–0.257	0.192–0.247	
Spleen	Mean	0.237 ^im^	0.203 ^i^	
	Sd	0.100	0.124	0.49
	Range	0.136–0.462	0.067–0.435	
	25–95%CI	0.245–0.29	0.219–0.317	
Liver	Mean	0.388 ^bdfjklm^	0.309 ^bdfj^	
	Sd	0.109	0.121	0.14
	Range	0.17–0.616	0.071–0.425	
	25–95%CI	0.397–0.446	0.324–0.421	

w.w.—wet weight. lowercase letters—the same lowercase letters indicate statistically significant differences at *p* < 0.05 between animal tissues within a one group of animals: normal and deficiency selenium level.

**Table 5:** Se concentration in individual tissues in heifers.

Tissue		Se Concentration (µg/g w.w.) in Heifers		Anova *p* Value
		Optimal Se Level	Deficient Se Status	
Serum μg/mL	Mean	0.107 ^ab^	0.063 ^ab^	
	Sd	0.017	0.013	<0.01
	Range	0.086–0.127	0.033–0.077	
	25–95%CI	0.108–0.124	0.070–0.083	
Longissimus dorsi muscle	Mean	0.095 ^cd^	0.101 ^cd^	
	Sd	0.036	0.048	0.84
	Range	0.028–0.205	0.044–0.182	
	25–95%CI	0.111–0.171	0.101–0.130	
Semitendinosus muscle	Mean	0.100 ^ef^	0.076 ^ef^	
	Sd	0.023	0.043	0.39
	Range	0.074–0.133	0.03–0.158	
	25–95%CI	0.101–0.122	0.085–0.111	
Kidney	Mean	1.473 ^aceghij^	1.383 ^aceghij^	
	Sd	0.570	0.366	0.38
	Range	0.96–2.47	0.879–1.929	
	25–95%CI	1.637–2.16	1.376–1.620	
Lungs	Mean	0.212 ^g^	0.161 ^g^	
	Sd	0.106	0.063	0.12
	Range	0.118–0.364	0.11–0.273	
	25–95%CI	0.242–0.340	0.164–0.201	
Heart	Mean	0.192 ^h^	0.176 ^h^	
	Sd	0.087	0.071	0.36
	Range	0.107–0.31	0.1–0.293	
	25–95%CI	0.219–0.297	0.176–0.219	
Spleen	Mean	0.260 ^i^	0.270 ^i^	
	Sd	0.134	0.116	0.83
	Range	0.125–0.456	0.127–0.433	
	25–95%CI	0.294–0.422	0.272–0.341	
Liver	Mean	0.420 ^bdfj^	0.230 ^bdfj^	
	Sd	0.137	0.149	0.02
	Range	0.278–0.652	0.093–0.59	
	25–95%CI	0.454–0.585	0.254–0.342	

w.w.—wet weight. lowercase letters—the same lowercase letters indicate statistically significant differences at *p* < 0.05 between animal tissues within a one group of animals: normal and deficiency selenium level.

**Table 6:** Se concentration in bulls, heifers and cows.

Tissue	Se Concentration (µg/g w.w.)		
	Bulls	Heifers	Cows
Serum μg/mL			
Mean	0.106 ± 0.034 ^abcd^	0.077 ± 0.028 ^a^	0.086 ± 0.050 ^a^
Longissimus dorsi muscle			
Mean	0.120 ± 0.04 ^aefgh^	0.102 ± 0.051	0.09 ± 0.049
Semitendinosus muscle			
Mean	0.101 ± 0.029 ^bei^	0.083 ± 0.037	0.09 ± 0.047 ^b^
Kidney			
Mean	1.560 ± 0.421 ^fj^	1.490 ± 0.34 ^b^	1.221 ± 0.410
Lungs			
Mean	0.224 ± 0.092 ^cgk^	0.189 ± 0.084 ^bcde^	0.156 ± 0.07
Heart			
Mean	0.218 ± 0.057 ^dhijk^	0.188 ± 0.073 ^cfg^	0.177 ± 0.085 ^a^
Spleen			
Mean	0.227 ± 0.106 ^l^	0.261 ± 0.124 ^dfh^	0.227 ± 0.104
Liver			
Mean	0.364 ± 0.116 ^l^	0.306 ± 0.171 ^aegh^	0.293 ± 0.169 ^b^

w.w.—wet mass. lowercase letters—the same lowercase letters indicate statistically significant differences at *p* < 0.05 between animal organs within a group.

**Table 7:** Correlation coefficient between serum and individual organs within a group.

	Longissimus Dorsi Muscle	Semitendinosus Muscle	Kidney	Lungs	Heart	Spleen	Liver
All animals							
Optimal Se level	−0.438 *	−0.218	−0.139	−0.113	−0.048	−0.402 *	−0.028
Deficient Se status	0.601 *	0.615 *	0.561 *	0.597 *	0.573 *	0.451 *	0.458 *
Cows							
Optimal Se level	−0.500	−0.022	0.238	0.563	0.594	−0.233	0.055
Deficient Se status	0.270	0.706 *	0.339	0.519	0.671 *	0.385	0.377
Bulls							
Optimal Se level	−0.388	−0.212	−0.133	−0.239	−0.287	−0.733 *	−0.040
Deficient Se status	0.723	0.756 *	0.599	0.825 *	0.932 *	0.436	0.914 *
Heifers							
Optimal Se level	0.134	0.169	−0.439	−0.386	−0.257	−0.378	0.049
Deficient Se status	−0.055	0.272	0.651	0.284	0.341	0.294	0.166

*—statistically significant differences at *p* < 0.05 between serum and tissues animal within a group.

## Data Availability

The data presented in this study are available on request from the corresponding author.

## Supplementary Materials

The following supporting information can be downloaded at: https://www.mdpi.com/article/10.3390/ani13243885/s1, Table S1. Correlation between analysed tissues of animals with normal Se status using Pearson R Correlation. Table S2. Correlation between analysed tissues of animals with deficient Se status using Pearson R Correlation. Table S3. Correlation between analysed tissues of cow with normal Se status using Pearson R Correlation. Table S4. Correlation between analysed tissues of cow with deficient Se status using Pearson R Correlation. Table S5. Correlation between analysed tissues of bulls with normal Se status using Pearson R Correlation. Table S6. Correlation between analysed tissues of bulls with deficient Se status using Pearson R Correlation. Table S7. Correlation between analysed tissues of heifers with normal Se status using Pearson R Correlation. Table S8. Correlation between analysed tissues of heifers with deficient Se status using Pearson R Correlation.
