# Supplementary material for: Total Selenium Level and Its Distribution between Organs in Beef Cattle in Different Selenium Status
Source: Animals (Basel). 2023 Dec 18;13(24):3885. doi: 10.3390/ani13243885 (PMC10740525; doi:10.3390/ani13243885)
Supplement: Supplementary file 1 [file animals-13-03885-s001.zip › animals-2737968-supplementary.pdf]

Table S1. Correlation between analysed tissues of animals with normal Se status using Pearson R Correlation

Tissue	Serum	The longest back muscle	Semitendegen muscle	Kidney	Lungs	Heart	Spleen	Liver
**Serum**		-0.438	-0.218	-0.139	-0.113	-0.048	-0.402	-0.028
**The longest back muscle**	*		0.490	0.249	0.210	0.121	0.349	0.235
**Semitendegen muscle**		*		0.148	0.260	-0.269	0.121	0.241
**Kidney**					0.080	0.012	0.331	0.409
**Lungs**						0.146	0.197	0.175
**Heart**							0.336	-0.333
**Spleen**	*							0.334
**Liver**				*				

* - Correlation statistically significant differences at p<0.05 between organ.

Table S2. Correlation between analysed tissues of animals with deficient Se status using Pearson R Correlation

Tissue	Serum	The longest back muscle	Semitendegen muscle	Kidney	Lungs	Heart	Spleen	Liver
**Serum**		0.601	0.615	0.561	0.597	0.573	0.451	0.458
**The longest back muscle**			0.437	0.341	0.489	0.345	0.260	0.179
**Semitendegen muscle**				0.039	0.419	0.381	0.211	0.277
**Kidney**					0.378	0.432	0.338	0.509
**Lungs**						0.838	0.450	0.756
**Heart**							0.512	0.820
**Spleen**								0.459
**Liver**								

* - Correlation statistically significant differences at p<0.05 between organ.

Table S3. Correlation between analysed tissues of cow with normal Se status using Pearson R Correlation

Tissue	Serum	The longest back muscle	Semitendegen muscle	Kidney	Lungs	Heart	Spleen	Liver
**Serum**		0.285	0.652	0.450	0.615	0.493	0.112	0.404
**The longest back muscle**			0.600	-0.064	0.222	0.288	0.451	0.618
**Semitendegen muscle**	*	*		0.300	0.679	0.365	0.505	0.657
**Kidney**	*				0.413	0.268	0.294	0.187
**Lungs**	*		*			0.536	0.447	0.640
**Heart**	*				*		0.510	0.314
**Spleen**		*	*		*	*		0.413
**Liver**		*	*		*			

* - Correlation statistically significant differences at p<0.05 between organ.

Table S4. Correlation between analysed tissues of cow with deficient Se status using Pearson R Correlation

Tissue	Serum	The longest back muscle	Semitendegen muscle	Kidney	Lungs	Heart	Spleen	Liver
**Serum**		0.270	0.706	0.339	0.519	0.671	0.385	0.377
**The longest back muscle**			0.650	-0.209	0.726	0.698	0.685	0.774
**Semitendegen muscle**	*			0.107	0.764	0.911	0.754	0.762
**Kidney**					0.031	0.141	0.228	0.162
**Lungs**		*	*			0.945	0.848	0.829
**Heart**	*	*	*		*		0.866	0.851
**Spleen**		*	*		*	*		0.843
**Liver**		*	*		*	*	*	

* - Correlation statistically significant differences at p<0.05 between organ.

Table S5. Correlation between analysed tissues of bulls with normal Se status using Pearson R Correlation

Tissue	Serum	The longest back muscle	Semitendegen muscle	Kidney	Lungs	Heart	Spleen	Liver
**Serum**		-0.388	-0.212	-0.133	-0.239	-0.287	-0.733	-0.040
**The longest back muscle**			0.682	0.580	0.319	0.572	0.287	-0.079
**Semitendegen muscle**		*		0.311	0.329	0.307	0.091	0.048
**Kidney**		*			0.016	0.425	0.274	0.002
**Lungs**						0.349	-0.013	-0.182
**Heart**		*					0.146	-0.452
**Spleen**	*							0.476
**Liver**								

* - Correlation statistically significant differences at p<0.05 between organ.

Table S6. Correlation between analysed tissues of bulls with deficient Se status using Pearson R Correlation

Tissue	Serum	The longest back muscle	Semitendegen muscle	Kidney	Lungs	Heart	Spleen	Liver
**Serum**		0.723	0.756	0.599	0.825	0.932	0.436	0.914
**The longest back muscle**			0.602	0.624	0.899	0.810	0.423	0.768
**Semitendegen muscle**	*			0.376	0.551	0.812	0.032	0.833
**Kidney**					0.546	0.657	0.386	0.662
**Lungs**	*	*				0.894	0.326	0.689

Tissue	Serum	The longest back muscle	Semitendegen muscle	Kidney	Lungs	Heart	Spleen	Liver
**Heart**	*	*	*		*		0.209	0.851
**Spleen**								0.498
**Liver**	*	*	*				*	

* - Correlation statistically significant differences at p<0.05 between organ.

Table S7. Correlation between analysed tissues of heifers with normal Se status using Pearson R Correlation

**Tissue**	**Serum**	**The longest back muscle**	**Semitendegen muscle**	**Kidney**	**Lungs**	**Heart**	**Spleen**	**Liver**
**Serum**		0.134	0.169	-0.341	-0.386	-0.257	-0.378	0.049
**The longest back muscle**			0.405	0.351	0.667	0.566	0.597	0.505
**Semitendegen muscle**				0.014	0.168	-0.042	0.313	0.192
**Kidney**					0.713	0.648	0.668	0.276
**Lungs**						0.948	0.975	0.740
**Heart**					*		0.911	0.839
**Spleen**					*	*		0.783
**Liver**						*	*	

* - Correlation statistically significant differences at p<0.05 between organ.

Table S8. Correlation between analysed tissues of heifers with deficient Se status using Pearson R Correlation

**Tissue**	**Serum**	**The longest back muscle**	**Semitendegen muscle**	**Kidney**	**Lungs**	**Heart**	**Spleen**	**Liver**
**Serum**		0.124	0.591	0.442	0.403	0.529	0.553	0.381
**The longest back muscle**			0.165	0.207	0.478	0.211	0.462	-0.066
**Semitendegen muscle**				0.549	0.129	-0.050	0.234	0.043
**Kidney**					0.624	0.035	0.621	0.272
**Lungs**						0.563	0.666	0.718
**Heart**							0.670	0.764
**Spleen**								0.494
**Liver**						*	*	

* - Correlation statistically significant differences at p<0.05 between organ.
